# AI in High-Frequency Micro-Ultrasound: Advancing Prostate Imaging from Segmentation to Cancer Detection

**DOI:** 10.3390/cancers18040665

**Published:** 2026-02-18

**Authors:** Ludovica Cella, Marco Paciotti, Pier Paolo Avolio, Vittorio Fasulo, Andrea Piccolini, Rebecca Canneto, Giacomo Cavadini, Luca Di Stefano, Alberto Saita, Paolo Casale, Massimo Lazzeri, Nicolò Maria Buffi, Giovanni Lughezzani

**Affiliations:** 1 Department of Biomedical Sciences, Humanitas University, Via Rita Levi Montalcini 4, Pieve Emanuele, 20072 Milan, Italy; 2 Department of Urology, IRCCS Humanitas Research Hospital, Via Manzoni 56, Rozzano, 20089 Milan, Italy; 3 Medical School, Humanitas University, Via Rita Levi Montalcini 4, Pieve Emanuele, 20072 Milan, Italy

**Keywords:** micro-ultrasound, artificial intelligence, deep learning, prostate cancer, image segmentation, cancer detection

## Abstract

Background/Objective: High-frequency micro-ultrasound (micro-US) offers real-time, high-resolution imaging for prostate cancer. Although artificial intelligence (AI) has shown potential in enhancing micro-US interpretation, a comprehensive review of this emerging field is currently missing. This review synthesizes current evidence on AI applied to ExactVu 29 MHz micro-US for prostate cancer. Methods: PubMed/MEDLINE, Embase, Scopus, Web of Science and the Cochrane Library were searched up to December 2025. Studies were included if they applied machine learning or deep learning directly to 29 MHz micro-US data and reported quantitative performance metrics. Results: Ten studies met the inclusion criteria: six on prostate cancer detection, three on prostate segmentation and one on micro-US–histopathology registration. Detection models ranged from classical quantitative ultrasound machine learning to deep architectures using self-supervision, transformers, multiple-instance learning, ensemble calibration and 3D segmentation-based pipelines. Among core-level models for clinically significant cancer, area under the receiver operating characteristic curve (AUROC) values clustered around 0.76–0.81; one lesion-level framework reported an AUROC of 0.92, though at a non-comparable analytical unit. Segmentation studies achieved accurate prostate delineation (Dice similarity coefficient ≈ 0.94), and a single study demonstrated high-precision 3D registration to whole-mount histopathology (Dice similarity coefficient 0.97 and landmark error < 3 mm). All studies evaluated AI on previously acquired data, without real-time clinical implementation. Conclusions: AI for micro-US shows promising and reproducible early results across detection, segmentation and registration, but evidence is still limited. In view of the potential of AI to optimize micro-US utilization and its related advantages, additional efforts are warranted to achieve clinical adoption.

## Simple Summary

High-frequency micro-ultrasound is an emerging imaging technique for prostate cancer that allows doctors to visualize prostate tissue in real time during biopsy. In recent years, artificial intelligence has been applied to micro-ultrasound images to help identify suspicious areas, outline the prostate gland, and improve biopsy targeting. However, the rapid growth of this field has made it difficult for clinicians to understand what these technologies can currently do and what their real limitations are. In this review, we summarize and critically assess all published studies that have applied artificial intelligence to 29 MHz ExactVu micro-ultrasound of the prostate. We describe how these systems are used for cancer detection, prostate segmentation, and image alignment, and we highlight the main technical and clinical challenges that still need to be addressed. This work provides a practical overview for clinicians and researchers and helps guide future development of artificial intelligence in prostate imaging.

## 1. Introduction

Artificial intelligence (AI) has rapidly reshaped diagnostic pathways, evolving from experimental algorithms into clinically relevant tools [1]. In radiology, pathology and oncology, deep learning models have achieved performance comparable to, and in some settings exceeding, that of human experts, supporting their progressive integration into diagnostic workflows [2,3,4]. Within urology, this evolution is particularly timely: while prostate cancer remains one of the most common malignancies among men worldwide, an appropriate balance between accurate early detection and avoidance of overdiagnosis is still lacking. Achieving this balance is essential for optimal risk stratification, treatment selection, and ultimately reducing the mortality burden [5].

In parallel, high-frequency micro-ultrasound (micro-US) has emerged as a disruptive imaging modality for prostate cancer [6,7,8]. Operating at approximately 29 MHz, micro-US provides a spatial resolution nearly three times higher than conventional transrectal ultrasound, enabling visualization of prostatic microarchitecture and real-time guidance during targeted biopsy [9,10]. Clinical evidence from both retrospective studies and a randomized clinical trial recently suggests that micro-US may offer a diagnostic performance comparable to that of multiparametric MRI (mpMRI), the current reference standard. Although micro-US also provides advantages in accessibility, cost, and procedural workflow, its widespread adoption has been limited by operator dependence and a steep learning curve, which hinder consistent image interpretation and reliable lesion targeting [11,12,13].

AI has the potential to mitigate these limitations by enabling objective, reproducible, and quantitative analysis of micro-US data. Reported applications span several domains, including automated segmentation of the prostate and peri-prostatic structures, lesion classification and prostate cancer detection, biopsy guidance through heatmaps or attention mechanisms, and uncertainty quantification to improve confidence and reduce false positives [14,15,16,17,18,19,20,21,22,23]. Over the last few years, small but growing multicenter efforts, together with advances in self-supervised, transformer-based and hybrid learning strategies, have contributed to a rapidly expanding, yet methodologically heterogeneous body of evidence [14,15,16,17,18,19,20,21,22,23].

To date, however, no review has specifically examined how AI is being applied to micro-US for prostate imaging. As a result, the field currently lacks a structured framework to compare studies that differ substantially in methodology, input data, and outcome definitions, making it difficult for clinicians to interpret reported performance and design future studies that address existing shortcomings. For these reasons, we present the first narrative review of AI applied to micro-US, aiming to synthesize the available evidence across (1) prostate cancer detection, (2) segmentation and (3) registration, critically appraise methodology and reported performance, and identify the gaps that must be addressed for clinical translation.

## 2. Materials and Methods

### 2.1. Search Strategy

A comprehensive literature search was conducted in PubMed/MEDLINE, Embase, Scopus, Web of Science and the Cochrane Library, without date restrictions. The last search was performed on December 2025. The search strategy combined controlled vocabulary (MeSH/Emtree) and free-text terms related to “micro-ultrasound”, “high-frequency ultrasound”, “prostate cancer”, “artificial intelligence”, “machine learning” and “deep learning”, using appropriate Boolean operators. Reference lists of eligible articles were also screened to identify additional relevant studies.

### 2.2. Study Selection and Eligibility Criteria

Given the rapid evolution and methodological diversity of AI applications to high-frequency micro-US, a narrative review was chosen as the most appropriate synthesis approach. Rather than aiming for quantitative pooling, our primary goal was to clarify the current state of the field and to provide a structured framework that clinicians can use to interpret the available results.

Studies were considered eligible if they (1) involved men undergoing high-frequency micro-US of the prostate, (2) applied AI, machine learning or deep learning methods directly to micro-US data, and (3) reported quantitative performance metrics (such as area under the receiver operating characteristic curve [AUROC], sensitivity, specificity, Dice coefficient or registration error) sufficient to allow comparison across studies.

Only peer-reviewed original research articles and full conference papers were included. Non-original articles, preprints, unpublished manuscripts, conference abstracts, studies not involving micro-US or AI methods, and papers without extractable quantitative data were excluded. Two reviewers (L.C. and M.P.) independently screened titles, abstracts and full texts, resolving disagreements by consensus. For transparency, the study selection process is summarized in a flow diagram provided in the Figure S1.

Critical appraisal was performed narratively by evaluating the comparability of analytical units and outcome metrics across studies, assessing dataset independence and external validity, and contextualizing findings within the broader AI literature in prostate imaging.

### 2.3. Data Extraction and Synthesis

Two reviewers (L.C. and M.P.) independently extracted key information from each included study, covering bibliographic details (author, year and country), study design and setting, dataset characteristics and imaging system, the AI approach (model type, input data, outputs and validation strategy), the reference standard (e.g., biopsy or whole-mount histopathology, expert image interpretation or paired imaging for registration tasks), and the reported performance metrics for classification, segmentation and registration tasks.

Findings were synthesized narratively and summarized in tables. Given the heterogeneity of analytical units (cores, regions of interest [ROIs], slices or lesions) and outcome metrics, the results were compared qualitatively, and segmentation or registration metrics were not interpreted as diagnostic performance. When metrics were sufficiently comparable, results were contrasted across studies and grouped by application (clinically significant prostate cancer [csPCa]/PCa detection, segmentation and registration), to highlight consistencies, discrepancies and remaining gaps in the literature that are most relevant for clinical translation.

## 3. Results

### 3.1. Study Selection and Characteristics

We identified ten original studies evaluating AI applied to high-frequency micro-US of the prostate [14,15,16,17,18,19,20,21,22,23]. Six addressed csPCa/PCa detection [14,15,16,17,19,23], three evaluated automated segmentation of the prostate or peri-prostatic structures [18,21,22], and one evaluated micro-US–histopathology image registration [20]. All studies were retrospective; four used multicenter cohorts [15,16,17,19], whereas the remaining six relied on single-center datasets [14,18,20,21,22,23]. The articles were published between 2018 and 2025 and all evaluated the ExactVu 29 MHz micro-US platform.

### 3.2. AI for Prostate Cancer Detection

Six studies evaluated AI-based models for prostate cancer detection using high-frequency micro-US. Of these, four studies specifically targeted clinically significant prostate cancer (csPCa, defined as Grade Group ≥ 2) as the primary endpoint, while the remaining two (Pensa et al. and Zhou et al.) targeted any prostate cancer [14,15,16,17,19,23]. Three were based on independent single-center datasets [14,15,23], while three studies used data from the same multicenter clinical trial (NCT02079025) [16,17,19]. Notably, these three studies draw from the same parent cohort with progressive expansions (578 to 693 patients); however, the degree of patient overlap between Gilany et al. and the later studies, as well as whether identical train/test splits were used, is not explicitly reported in the original publications.

Across these works, four analytical units were employed: (1) core-level, (2) slice-level, (3) lesion-level, and (4) patient-level detection (Table 1). In several studies, region of interest (ROI)-level classification was used as an intermediate processing step (extracting and classifying small patches of ultrasound data) but ROI-level predictions were always aggregated to a higher-level unit for final evaluation and were not used as a standalone analytical output.

Core-level models predict the presence of csPCa in individual biopsy cores. Because each core is independently submitted for pathological analysis and reported as positive or negative for csPCa, this analytical unit aligns closely with how histopathology is reported and biopsies are performed, making AI predictions directly interpretable in the context of biopsy decision-making [15,16,17,19].

In Rohrbach et al. and Wilson et al., ROI-level features or predictions were explicitly aggregated (via summary statistics or averaging) to produce core-level outputs [15,19].

Slice-level detection operates on reconstructed 2D micro-US slices extracted from 3D sweeps and reports whether cancer is present anywhere within the cross-sectional plane. This unit was used by Pensa et al. [14].

Lesion-level detection involves segmenting suspicious 3D regions on micro-US volumes and evaluating whether each predicted lesion overlaps with a biopsy-confirmed cancer focus. This unit was used by Zhou et al. (ProMUS-NET) [23].

Patient-level detection evaluates whether cancer is correctly identified in a given patient, regardless of the number or location of individual lesions. Zhou et al. [23] also reported patient-level metrics as a secondary outcome, providing a clinically intuitive measure of overall diagnostic performance.

Performance metrics (AUROC, sensitivity, and specificity) are therefore reported at different levels across studies and should be interpreted within the corresponding analytical unit.

At the core level, Rohrbach et al. analyzed 1956 biopsy cores from 163 patients across five centers using classical machine-learning classifiers trained on quantitative ultrasound features derived from radiofrequency data, reporting AUROC values of 0.77–0.81 for csPCa detection [15]. Gilany et al. trained a self-supervised CNN feature extractor with a transformer-based multiple-instance learning (MIL) aggregator on data from 578 patients across five centers, achieving a core-level AUROC of 0.803 [16]. Using the same multicenter cohort expanded to 693 patients (6607 total cores), Wilson et al. benchmarked several uncertainty estimation methods for convolutional models trained on ROI-level patches and aggregated to core-level predictions, reporting AUROC values up to 0.76 with a 10-fold cross-validation scheme [19]. Harmanani et al. combined self-supervision, MIL, and deep ensembles with random-undersampled boosting on the same 693-patient dataset, obtaining a core-level AUROC of 0.799 [17] (Table 2).

At the slice level, Pensa et al. co-registered 3D micro-US sweeps to whole-mount prostatectomy specimens from 15 patients, generating 977 reconstructed 2D sagittal images. ConvNeXt-based models fine-tuned via transfer learning classified whether each slice contained cancer, with the best ensemble model yielding a sensitivity of 78.9%, specificity of 72.7%, and AUROC of 0.802 on a three-patient test set [14].

At the lesion level, Zhou et al. (ProMUS-NET) trained a 3D nnU-Net on micro-US image stacks from 64 patients, with 51 GG ≥ 2 lesions annotated by cross-referencing MRI and biopsy pathology information. The model produced voxel-level probability maps that were post-processed into discrete lesion predictions, achieving a lesion-level AUROC of 0.92 and a lesion-level sensitivity of 73%, compared with 58% for expert urologists in a matched sub-cohort. Patient-level sensitivity was 77% for the AI model vs 66% for urologists. However, patient-level specificity was zero (meaning the model flagged at least one suspicious lesion in every patient without csPCa on biopsy) compared with 39% for urologists. Lesion-level specificity was 95%, indicating that while the model rarely misclassified individual prostate sectors, it consistently generated at least one false-positive prediction per patient [23].

Performance metrics are meaningful only when compared *within* the same analytical unit. Cross-unit comparisons are inappropriate and can mislead readers into overestimating or underestimating model performance.

### 3.3. AI for Prostate Segmentation

Three studies addressed automated segmentation of the prostate and peri-prostatic structures on micro-US, all employing encoder–decoder architectures with transformer or attention components optimized for high-frequency ultrasound [18,21,22] (Table 3).

All three studies utilized the same 75-patient single-center dataset for model development and evaluation, raising important concerns about generalizability and the independence of reported performance metrics.

The architectures differed primarily in their strategies for feature integration and spatial modeling. MicroSegNet employed a TransUNet-based framework with multi-scale supervision and an annotation-guided loss function designed to focus learning on regions where expert and non-expert annotations diverged, achieving a Dice similarity coefficient of 0.939 and Hausdorff distance (HD95) of 2.02 mm [18]. HEFFLPNet extended this approach by incorporating hierarchical feature-fusion modules and multi-scale prediction attention, yielding comparable performance (Dice 0.938, HD95 2.12 mm) on the same dataset. Importantly, HEFFLPNet was also evaluated on conventional transrectal ultrasound images, demonstrating reasonable cross-modality generalization despite the substantial domain shift; however, this does not substitute for independent external validation [21]. Al-Qurri et al. introduced a more complex architecture combining dual CNN-Transformer encoders with a Mamba-based decoder and hypergraph neural network for modeling higher-order spatial relationships, achieving marginally higher metrics (Dice 0.942, HD95 1.93 mm) [22].

Despite architectural differences ranging from relatively straightforward multi-scale supervision to elaborate hypergraph-based spatial modeling, all three approaches converged on remarkably similar performance levels (Dice 0.938–0.942, HD95 1.93–2.12 mm).

### 3.4. AI for Image Registration (Micro-US–Histopathology)

One proof-of-concept study evaluated deep-learning-based registration between in vivo micro-US and ex vivo pseudo–whole-mount histopathology images. Imran et al. developed a semi-automated pipeline for 18 radical prostatectomy patients that reconstructed 3D micro-US volumes from oblique acquisitions and digitally stitched histology fragments [20]. Their two-stage registration framework (combining ResNet-18-based affine transformation with a U-Net-style deformable registration network) achieved high accuracy with a Dice coefficient of 0.971, mean Hausdorff distance of 2.02 mm, and mean landmark error of 2.84 mm. This approach successfully mapped pathologist-annotated cancer outlines from histopathology onto micro-US images, creating a validated dataset for training diagnostic models and improving clinical interpretation of micro-US.

## 4. Discussion

Given the potential of AI to enhance micro-US within the prostate cancer diagnostic pathway, this review summarizes and critically appraises current evidence on AI for 29 MHz micro-US. It provides a structured synthesis across detection, segmentation and registration tasks, highlighting both technical advances and unresolved limitations.

Across these domains, available evidence indicates that 29 MHz micro-US contains rich quantitative information that can be exploited by AI models. However, all ten included studies relied on retrospective, offline analyses, and no system has yet been evaluated in real time during clinical examinations or targeted biopsy procedures [14,15,16,17,18,19,20,21,22,23] (Table 4).

A key source of heterogeneity across the included studies is the choice of analytical unit. Core-level models predict the presence of csPCa in individual biopsy cores and align closely with how histopathology is reported and biopsies are performed. Slice-level and lesion-level approaches address different clinical questions—image-based screening and spatial lesion localization, respectively—and their performance metrics are not directly comparable to core-level AUROCs.

The included detection studies span a broad methodological spectrum, from classical machine-learning classifiers trained on hand-crafted quantitative ultrasound features [15] to self-supervised transformers with MIL aggregation [16] and uncertainty-aware deep ensembles [17,19]. Notably, although Wilson et al. and Harmanani et al. incorporated calibration or uncertainty estimation into their frameworks, formal calibration metrics (e.g., expected calibration error) were not consistently reported across all detection studies, limiting the assessment of prediction reliability [17,19]. Despite these architectural differences, core-level AUROC values remain tightly clustered between 0.76 and 0.81. This narrow range is notable: models built on very different principles converge on essentially the same diagnostic accuracy. One plausible interpretation, which remains to be validated in future studies, is that current performance may be limited not only by the choice of algorithm, but also by the amount of diagnostic information that 29 MHz micro-US images inherently contain. If this is the case, meaningful improvements may require richer input data—for example, integrating clinical variables, MRI, or higher-quality ground-truth labels—rather than further model refinement alone.

ProMUS-NET expanded the task to three-dimensional lesion segmentation, achieving a lesion-level AUROC of 0.92 [23]. However, this metric reflects a different analytical unit and is not directly comparable to core-level classifiers. ProMUS-NET also showed zero patient-level specificity, highlighting the trade-off between sensitivity and clinical usability. It remains the only detection study reporting patient-level metrics, whereas all others are limited to core- or slice-level evaluation.

In the domain of segmentation, three studies evaluated automated delineation of the prostate capsule on micro-US, all relying on encoder–decoder architectures [18,21,22]. MicroSegNet combines convolutional layers with transformer blocks for contextual information; HEFFLPNet uses attention-guided fusion to emphasize the most informative features; and Al-Qurri et al. proposed a hybrid CNN-Transformer dual encoder with a Mamba-based decoder and hypergraph reasoning. All three studies, however, used the same 75-patient single-center dataset, raising concerns about whether reported improvements reflect genuine architectural advances or overfitting to the same test distribution. The narrow spread of performance across methods (Dice 0.938–0.942 and HD95 1.93–2.12 mm) suggests a ceiling that may be determined by the quality and consistency of manual annotations rather than by model refinement. However, none of these studies formally quantified inter-observer annotation variability or evaluated segmentation performance during live scanning.

Evidence in the area of image registration is currently limited to a single proof-of-concept study. Imran et al. demonstrated that deep neural networks can align in vivo micro-US volumes with ex vivo pseudo–whole-mount histopathology, achieving excellent prostate overlap (Dice 0.97) and low landmark error (~2.8 mm) [20]. Such frameworks are essential for generating anatomically precise lesion labels and may accelerate future large-scale ground-truth creation, currently a major bottleneck in the field.

Several cross-cutting limitations emerge from the available evidence.

First, no study has validated AI performance in real time. All models were trained and tested on static datasets, without assessment of latency under clinical conditions, probe motion, anatomical deformation, or operator variability.

Second, the heterogeneity and imperfection of ground truth remains a fundamental unresolved challenge. Reference standards range from targeted biopsy cores, which sample only a small fraction of the prostate and may miss csPCa, to pseudo–whole-mount histopathology, which involves tissue deformation during processing, to true whole-mount sections, which are rarely available. Biopsy-based ground truth, used in most detection studies, has limited sensitivity and spatial precision; models trained on such labels may learn to reproduce these imperfections rather than detect true disease. This inconsistency undermines the comparability of reported metrics across studies.

Third, issues of external validity and generalizability remain essentially unaddressed. Most studies rely on small, single-center datasets, and several reuse overlapping patient cohorts from the same multicenter trial, reducing effective sample independence. Inter-operator variability, vendor-specific characteristics, and geographic diversity are largely untested despite micro-US being highly operator-dependent. Only MicroSegNet released its dataset publicly; all others rely on proprietary cohorts, limiting reproducibility.

From a clinical standpoint, AI-enhanced micro-US is promising but remains in an early translational phase. Core-level csPCa detection aligns most directly with biopsy decision-making, lesion-level segmentation could support targeted biopsy planning, and prostate segmentation is technically mature but offers incremental benefit unless embedded within navigation or fusion frameworks.

More broadly, AI can enhance micro-US along several key parameters: standardizing image interpretation across operators with different experience levels, replacing subjective visual scoring with quantitative probability estimates, and enabling real-time decision support through heatmaps that guide biopsy targeting, collectively addressing the operator dependence that currently limits micro-US adoption.

Compared with MRI, micro-US benefits from lower cost, shorter waiting times, and true real-time capability [24,25,26,27]. However, the maturity of AI development differs substantially between the two modalities: mpMRI-based AI operates on significantly larger, often multicenter datasets (e.g., the PI-CAI study included over 10,000 cases), benefits from standardized acquisition protocols (PI-RADS), has undergone rigorous external validation, and several tools have obtained regulatory approval [28,29,30,31]. In contrast, micro-US AI remains at a proof-of-concept stage, with smaller cohorts (largest multicenter study: 693 patients), less standardization, and no regulatory approvals. Despite increasing international visibility of micro-US, including evidence from a recent randomized trial [13], its real-world clinical penetration remains limited, largely because the technique is perceived as highly operator-dependent and insufficiently reproducible. AI may play a valuable role in addressing this limitation by reducing operator dependence and shortening the learning curve.

To the best of our knowledge, this is the first work to comprehensively synthesize AI applications to micro-US prostate imaging across all major analytical tasks. This review has limitations. As a narrative synthesis, it cannot provide pooled quantitative estimates, formal risk-of-bias assessment, or head-to-head comparisons. The small number of studies, their methodological heterogeneity, and the lack of publicly available datasets limit generalizability. Some studies were derived from partially overlapping patient cohorts, reducing the effective independence of the evidence. Furthermore, all included studies rely on the same commercial 29 MHz ExactVu platform, confining current evidence to a single system. These constraints underscore the need for standardized reporting, open datasets, and prospective multicenter evaluation. 

## 5. Conclusions

This study provides the first comprehensive review of AI applied to high-frequency micro-US for prostate imaging. Across six detection studies, AI models trained on 29 MHz micro-US consistently achieved core-level AUROC values of approximately 0.76–0.81 for clinically significant disease, while lesion-level detection reached an AUROC of 0.92, though at a different analytical unit. Prostate capsule segmentation can be performed with high spatial accuracy (Dice ≈ 0.94), and micro-US volumes can be registered to whole-mount histopathology with a sub-3 mm landmark error.

Despite these encouraging results, all available data are retrospective, largely vendor-specific and evaluated offline, with outcomes reported mainly at core level and without assessment of real-time performance or impact on clinical decision-making. Further progress will require larger multi-vendor and multicenter cohorts, standardized definitions and endpoints, and prospective, patient-level studies embedded in contemporary MRI- and micro-US-guided biopsy pathways. The development and validation of calibrated, interpretable models that can operate in real time within routine workflow constraints will be essential for AI-enhanced micro-US to transition from technical feasibility to clinically relevant implementation.

## 6. Future Directions

Future progress will depend on advances in three interconnected areas: technical validation, clinical validation, and clinical implementation.

On the technical side, multi-vendor validation is urgently needed; current evidence is confined entirely to the ExactVu 29 MHz platform. Real-time evaluation is equally critical: AI models must be tested during live scanning and biopsy to assess diagnostic accuracy, inference latency, robustness to probe motion and artifacts, and impact on procedure time and operator cognitive load. Underpinning both efforts, standardized data collection and reporting protocols must be established, including consensus definitions for ground truth, standardized data splits preventing leakage across overlapping cohorts, and mandatory reporting of calibration metrics and failure modes.

On the clinical side, prospective multicenter trials should report patient-level outcomes rather than core-, slice-, or lesion-level AUROCs alone. These trials should incorporate clinical utility analyses, including decision-curve analysis, to quantify net benefit across clinically relevant risk thresholds compared with standard mpMRI-based and biopsy-all pathways. Head-to-head comparisons with mpMRI within the same patient cohort are needed to establish whether micro-US AI can serve as a standalone pathway or functions best as a complementary tool where MRI is inaccessible.

Finally, work is needed to define how micro-US AI should be integrated into clinical practice. Decision-analytic and pathway modeling should clarify where the technology fits best—for example, as a first-line triage tool in intermediate-risk, biopsy-naïve men versus as an adjunct to mpMRI. In parallel, health-economic evaluation encompassing workflow time, throughput, cost per csPCa detected, and budget impact compared with mpMRI pathways will be essential to support adoption. Crucially, these systems should be designed and regulated as clinical decision-support tools, not autonomous diagnostic agents.

## Figures and Tables

**Table 1:** Analytical units in AI-based prostate cancer detection on micro-US.

Analytical Unit	What Is Classified	Clinical Question Addressed	Studies Using This Unit
Core-level	Individual biopsy core labeled benign vs clinically significant cancer (GG ≥ 2)	“Does this sampled tissue location contain csPCa?”	Gilany 2023 [16], Harmanani 2025 [17], Wilson 2024 [19], Rohrbach 2018 [15]
Slice-level	Reconstructed 2D image slice from a 3D sweep	“Does this cross-sectional plane contain cancer?”	Pensa 2024 [14]
Lesion-level	Segmented 3D suspicious lesion	“Is this volumetrically defined lesion malignant?”	Zhou 2025 [23]
Patient-level	Entire patient	“Does this patient have csPCa anywhere in the prostate?”	Zhou 2025 [23]

csPCa = clinically significant prostate cancer; ROI = region of interest; micro-US = high-frequency micro-ultrasound.

**Table 2:** Characteristics of the included studies for PCa detection using core-level.

Study	Year	Country	Study Design	Setting	Dataset	AI Method	AUROC
Rohrbach et al. [15]	2018	USA	Retrospective	Multicenter	163 pt, 1956 cores	Classical ML on QUS features	0.770–0.810
Gilany et al. (TRUSformer) [16]	2023	Canada	Retrospective	Multicenter	578 pt, 6607 cores (NCT02079025)	Self-supervised + MIL Transformer	0.803
Wilson et al. [19]	2024	Canada	Retrospective	Multicenter	693 pt, 6607 cores (NCT02079025)	CNN + calibration	0.760
Harmanani et al. (TRUSWorthy) [17]	2025	Canada	Retrospective	Multicenter	693 pt, 6607 cores (NCT02079025)	Self-supervision + MIL + calibrated ensemble	0.799

micro-US = high-frequency micro-ultrasound; TRUS = transrectal ultrasound; PCa = prostate cancer; csPCa = clinically significant prostate cancer; AI = artificial intelligence; ML = machine learning; DL = deep learning; pt = patients; CNN = convolutional neural network; QUS = quantitative ultrasound; RF = radiofrequency; MIL = multiple-instance learning; SSL = self-supervised learning; AUROC = area under the receiver operating characteristic curve. Note: Patient counts differ between Gilany et al. (578 patients) and the later studies by Wilson et al. and Harmanani et al. (693 patients) because the latter used an expanded version of the same NCT02079025 cohort; however, the total number of biopsy cores (6607) remained unchanged across reports.

**Table 3:** Characteristics of the included studies for prostate segmentation.

Study	Year	Country	Study Design	Setting	n Patients	Dataset Size	AI Method	Reference Standard	Dice	HD95 (mm)
Jiang et al. (MicroSegNet) [18]	2024	USA	Retrospective	Single-center	75	2060 train + 758 test images	TransUNet + multi-scale	Expert contours	0.939	2.02
Huang et al. (HEFFLPNet) [21]	2025	China	Retrospective	Single-center	75	2060 train + 758 test images	CNN + attention fusion	Expert contours	0.938	2.12
Al-Qurri et al. [22]	2025	USA	Retrospective	Single-center	75	2060 train + 758 test images	CNN-Transformer + Mamba	Expert contours	0.942	1.93

CNN = convolutional neural network; Dice = Dice similarity coefficient; HD95 = 95th percentile Hausdorff distance.

**Table 4:** Clinical relevance of AI applications in micro-US.

AI Task	What the AI Does	Clinical Relevance	Current Limitations
Cancer detection	Highlights image regions suspicious for clinically significant PCa	Guides targeted biopsy and focal sampling	Limited ground truth and lack of large prospective validation
Prostate segmentation	Delineates prostate boundaries	Enables anatomy identification and volume estimation	Lack of real-time deployment during live scanning
Image registration (micro-US to histology/MRI)	Maps histopathology or MRI onto micro-US	Enables spatially accurate ground truth and multimodal analysis	Tissue deformation and limited availability of matched radical prostatectomy datasets

AI: artificial intelligence; PCa: prostate cancer; micro-US: high-frequency micro-ultrasound; MRI: magnetic resonance imaging.

## Data Availability

No new data were created or analyzed in this study. Data sharing is not applicable to this article.

## Supplementary Materials

The following supporting information can be downloaded at: https://www.mdpi.com/article/10.3390/cancers18040665/s1, Figure S1. Flow diagram summarizing the literature search and study selection process.

## Abbreviations

The following abbreviations are used in this manuscript:

AI	Artificial intelligence
AUROC	Area under the receiver operating characteristic curve
CNN	Convolutional neural network
csPCa	Clinically significant prostate cancer
ML	Machine learning
mpMRI	Multiparametric magnetic resonance imaging
micro-US	High-frequency micro-ultrasound
PCa	Prostate cancer
ROI	Region of interest
TRUS	Transrectal ultrasound
MIL	Multiple-instance learning
QUS	Quantitative ultrasound
HD95	95th percentile Hausdorff distance
GG	Grade Group
PI-RADS	Prostate Imaging Reporting and Data System
DL	Deep learning
RF	Radiofrequency
SSL	Self-supervised learning
Pt	Patients
