# Supplementary figures and images for: AI in High-Frequency Micro-Ultrasound: Advancing Prostate Imaging from Segmentation to Cancer Detection
Source: Cancers (Basel). 2026 Feb 18;18(4):665. doi: 10.3390/cancers18040665 (PMC12939672; doi:10.3390/cancers18040665)

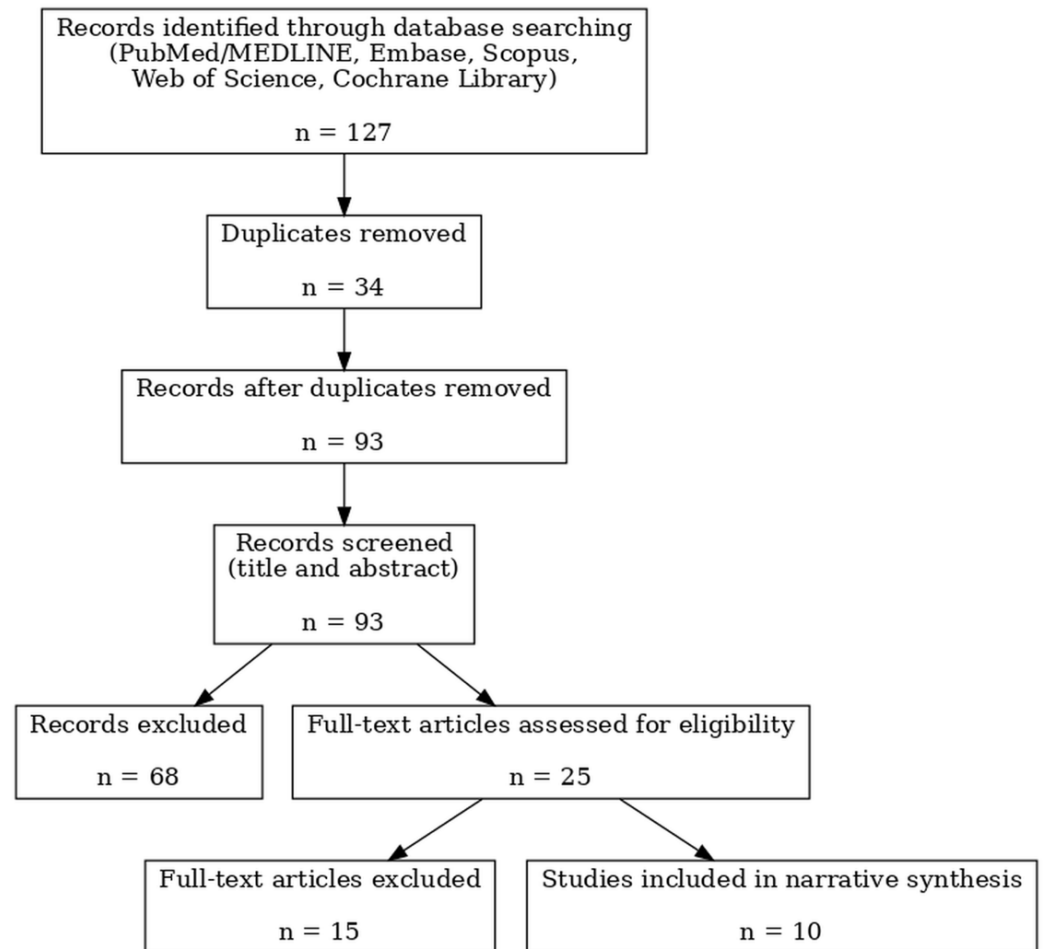

**Figure S1.** Flow diagram summarizing the literature search and study selection process.

Supplement: Supplementary file 1 [file cancers-18-00665-s001.zip › cancers-4123898-supplementary.pdf]
